# Utilization of Flow Cytometry, Metabolomic Analyses and a Feline Infectious Peritonitis Case Study to Evaluate the Physiological Impact of Polyprenyl Immunostimulant

**DOI:** 10.3390/cells14100752

**Published:** 2025-05-21

**Authors:** Irene Lee, Amar Desai, Akshay Patil, Yan Xu, Kelley Pozza-Adams, Anthony J Berdis

**Affiliations:** 1 Department of Chemistry, Case Western Reserve University, Cleveland, OH 44106, USA; 2 CASE Comprehensive Cancer Center, Case Western Reserve University, Cleveland, OH 44106, USA; abd10@case.edu; 3 Department of Chemistry, Cleveland State University, Cleveland, OH 44115, USA; patilakshaydr@gmail.com (A.P.); y.xu@csuohio.edu (Y.X.); a.berdis@csuohio.edu (A.J.B.); 4 Tremont Animal Clinic, 2885 W 25th St, Cleveland, OH 44113, USA; tremontclinic@gmail.com

**Keywords:** viral infection, immunomodulator, metabolomics, flowcytometry, immunotherapy, coronavirus

## Abstract

Measles, hepatitis C, and COVID-19 are significant human diseases caused by RNA viruses. While vaccines exist to prevent infections, there are a small number of currently available therapeutic agents that can effectively treat these diseases after infection occurs. This study explores a new therapeutic strategy using a small molecule designated polyprenyl immunostimulant (PI) to increase innate immune responses and combat viral infections. Using a multi-disciplinary approach, this study quantifies the effects of PI in mice and THP-1 cells using flow cytometry to identify immune phenotypic markers and mass spectroscopy to monitor the metabolomic profiles of immune cells perturbed by PI treatment. The metabolomic studies identified that sphinganine and ceramide, which are precursors of sphingosine-1-phosphate (S1P), were the common metabolites upregulated in THP-1 and mice blood. Sphingosine-1-phosphate can mediate the trafficking of T cells, whereas ceramide can signal the activation and proliferation of T cells, thereby modulating the mammalian host’s immunity. To demonstrate proof-of-principle, a case study was conducted to examine the benefit of administering PI to improve the outcomes of a feline co-infected with two distinct RNA viruses—feline leukemia virus and feline infectious peritonitis virus. Both viruses produce deadly symptoms that closely resemble RNA viruses that infect humans. The results identify quantifiable cellular and metabolic markers arising from PI treatment that can be used to establish a platform measuring the efficacy of PI in modulating the innate immune system.

## 1. Introduction

Coronaviruses (CoVs) are a family of enveloped viruses containing a positive-sense RNA genome that infect a broad range of vertebrates including mammals and birds [1,2,3,4]. While over 100 species of CoVs have been identified [5,6], only seven CoV strains have been isolated from humans [7,8,9,10]. Of these, four strains (HCoV-229E, HCoV-NL63, HCoV-HKU1, and HCoV-OC43) usually produce mild upper respiratory infections and account for an estimated 15–30% of all reported cases of the “common cold” [11]. However, the three most recently identified human CoV strains pose significantly greater health threats as they produce more serious respiratory infections. These include severe acute respiratory syndrome (SARS-CoV), identified in 2002; Middle East respiratory syndrome (MERS-CoV), identified in 2012; and SARS-CoV-2, identified in 2019 [12,13,14,15,16,17,18,19]. This most recent HCoV is by far the most significant as it has infected more than 700 million people and caused the death of more than 7 million people worldwide [20,21].

In addition to humans, CoVs infect a large number of mammalian and avian species to cause diseases in companion animals (cats and dogs) and livestock (cattle, pigs, and chickens) [2,4]. In this study, we used feline coronavirus (FCoV) as a model for human CoVs due to the striking similarities between the two [22]. There are also several relevant clinical similarities between SARS-CoV-2 and FCoV infections. For instance, humans infected with SARS-CoV-2 and felines infected with FCoV can be asymptomatic or display mild symptoms including fever, diarrhea, dehydration, and sneezing/coughing. In felines, however, persistent infection leads to mutational events that transform FCoV into a highly virulent strain called feline infectious peritonitis virus (FIPV) [23,24,25]. FIPV is macrophage tropic and is believed to cause aberrant cytokine and/or chemokine expression, which causes lymphocyte depletion. This culminates in a highly fatal disease called feline infectious peritonitis (FIP) and is similar to the “cytokine storm” reported in COVID-19 patients, which causes life-threatening symptoms such as pulmonary infiltrates and cardiovascular shock [26,27].

To date, there are few antiviral agents that can combat CoV infections. One notable exception is the nucleoside analog, Remdesivir (Gilead Science), which received emergency FDA approval in late 2020 to treat certain patients infected with SARS-CoV-2 [28]. When tested in cell culture systems, Remdesivir showed efficacy toward inhibiting SARS-CoV and MERS-CoV replication. A recent NIH clinical trial showed that Remdesivir treatment in SARS-CoV-2-infected patients reduced the median time to recovery from 11 days to 15 days [29]. Unfortunately, Remdesivir did not significantly improve mortality in patients infected with SARS-CoV-2. Another nucleoside analog, molnupiravir, is effective in treating mild to moderate COVID-19 infections in non-hospitalized patients. However, its effectiveness in treating late-stage or severe COVID-19 patients is unclear [30]. Furthermore, molnupiravir does not appear to reduce the risk of mortality and the hospitalization of vaccinated patients. These limitations highlight the urgent need to develop innovative therapeutic strategies and agents to treat severe CoV infections. Interestingly, remdesivir and molnupiravir have been successfully used to treat FIP in non-immune compromised felines within a treatment window of about 84 days, which is much longer than the treatment window for COVID-19 in humans [31].

An alternative to nucleoside analogs that inhibit viral replication is the development of therapeutic strategies that increase immune function. One attractive candidate is PI (VetImmune), which is a USDA-approved and commercially available small molecule used in veterinary medicine to treat feline rhinotracheitis (FRV) [32]. More recently, PI has been repositioned as a health supplement for boosting innate immunity in both felines and canines. PI is also used “off-label” to treat felines infected either with feline leukemia virus (FeLV, I. Lee unpublished results) or non-effusive FIP [33,34,35]. FeLV is an oncornavirus (RNA virus) of the family Retroviridae [36]. The median survival time for cats infected with FeLV is approximately 2.5 years. In this case, morality is typically caused by severe anemia, abnormal white blood cell counts, and compromised bone marrow function. Felines suffering from FIP and FeLV display similar hematological abnormalities and dysfunction. In addition, FIP induces a globally dysregulated inflammatory response that destroys several major organs, including the lungs, kidneys, and the central nervous system [37].

**Figure 1:**
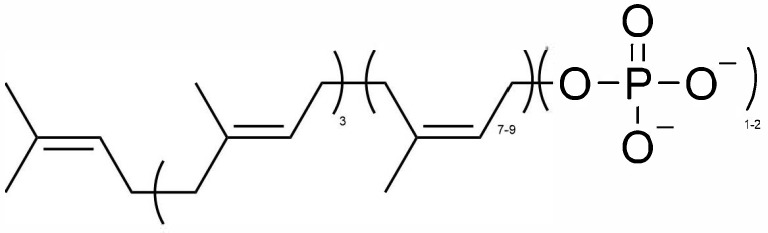
Chemical structure of polyprenylimmunostimulant (PI). 3 = number of isoprene units in trans-configurations; 7–9 = number of isoprene units in cis-configurations; 1–2 = number of phosphoric anions.

Unlike nucleoside analogs such as Remdesivir and Molnupiravir, PI is a chemical mixture of phosphorylated polyisoprenols containing three trans- and several cis-isoprene units (Figure 1). The antiviral efficacy of PI has been demonstrated in felines through analyses of clinically relevant data monitoring changes in the appetite/body weight, blood chemical profiles, and complete blood count values of infected felines [33,34,35]. This information shows that PI can cause remission of the non-effusive form of FIP. Despite this encouraging clinical data, the molecular mechanism of action of PI remains elusive. One cell-based study using the human cell line THP-1 showed the modest upregulation of TNFα levels within hours after PI treatment [38]. Flow cytometry analyses detected an elevated level of CD11b in whole blood and isolated monocytes in felines inoculated with FRV and treated with PI over 3 days. An elevated level of CD11b was attributed to an activated innate immune response [39]. Taken together, these results suggest that PI combats viral pathogenesis by reprogramming the cell-mediated innate immune response.

Since the symptoms of FIP resemble those found in human COVID-19 [40], we propose that PI can potentially treat COVID-19 and possibly other viral infections in humans. PI could synergize with an antiviral agent such as Molnupiravir to treat viral infections by directing the host’s immune system to actively remove the chemically inactivated viruses. However, more rigorous quantitative characterization of the cellular pathways modulated by PI must be performed to reposition PI as a human therapeutic agent. This study bridges this gap in knowledge using a pre-clinical mouse model combined with mammalian cell culture systems to characterize immunological outcomes in response to PI treatment.

## 2. Materials and Methods

### 2.1. Materials

Phosphate-buffered saline (PBS), antibiotic and antifungal agents, amphotericin, propidium iodide, PrestoBlue, DAPI, Alexa Fluor 488, and the apoptosis assay kit containing Alexa Fluor 488-labeled Annexin V were obtained from Invitrogen (ThermoFisher Scientific, Waltham, MA, USA) Polyprenyl immunostimulant (PI) was supplied by SASS & SASS, Inc. (Oak Ridge, TN, USA) as a solid and dissolved in HPLC grade n-butanol as a 10 mg/mL stock. LC-MS-grade acetonitrile and methanol were purchased from Thermo Fischer Scientific (Waltham, MA, USA) for metabolomic studies. Pure acetic acid (99.8) was purchased from Acros Organics, (ThermoFisher Scientific, Waltham, MA, USA) and Deionized water with a resistance of 18.2 MΩ was prepared using a Barnstead GenPure xCAD ultrapure water system from Thermo Fisher Scientific (Waltham, MA, USA). Ammonium acetate (LC-MS grade) was obtained from Sigma-Aldrich (St. Louis, MO, USA). Chloroform was purchased from Sigma-Aldrich (St. Louis, MO, USA). Prostaglandin E2-d4 (PGE2-d4) standard solution (500 μg/mL in methyl acetate, (>99% purity) was purchased from Cayman Chemical (Ann Arbor, MI, USA) and used as the internal standard (IS) master stock. THP-1 cells were obtained from ATCC (Manasses, VA, USA).

### 2.2. Methods

Cell culture: THP-1 cells were cultured in a humidified atmosphere of 5% CO_2_ at 37 °C. Cells were grown in RPMI (Sigma, St. Louis, MO, USA) supplemented with 10% fetal bovine serum (FBS) (Biowest, Lakewood Ranch, FL, USA) and 1.0% Penicillin Streptomycin (Gibco, ThermoFisher Scientific, Waltham, MA, USA) at 37 °C with 5.0% CO_2_. Since this cell line was obtained from ATCC, informed consent was not required. Cells were routinely authenticated based on their morphology and growth characteristics. All cells were expanded and then frozen at low passage (passages 2–5) within 2 weeks after the receipt of the original stocks. All cells used for experiments were between passages 6 and 12. Cell lines were tested for mycoplasma after each thaw or every 4 weeks when grown in culture. Mycoplasma infection was detected using the MycoAlert Mycoplasma Detection kit from Lonza (Walkersville, MD, USA).

**Cell viability assays:** THP-1 cells were plated at an initial density of 200,000 cells/mL. PI was added to wells in a dose-dependent manner (1−100 mg/mL). In all experiments, the final concentration of the co-solvent, n-butanol, was maintained at 0.1%. Cells were treated and assessed for viability for time periods ranging from 2 to 72 h. Cell viability was assessed using a Muse Cell Count (EMD Millipore, Burlington, MA, USA) and Cell-Titer blue assays.

**Apoptosis Measurements:** THP-1 cells were plated at an initial density of 200,000 cells/mL. Cells were treated with variable concentrations of PI, as described above. At variable time intervals (2–72 h), cells were harvested by centrifugation, washed in PBS, and re-suspended in 100 mL of binding buffer containing 5 mM of Annexin V-Alexa Fluor 488 conjugate. Cells were treated with 1 mg/mL propidium iodide and incubated at room temperature for 15 min, followed by flow cytometry analysis. Cells were analyzed using a Muse Cell analyzer. In total, 15,000-gated events were observed for each sample.

### 2.3. Animal Studies

CWRU Animals: Animals were housed in AAALAC-accredited facilities in the CWRU School of Medicine. The experimental procedures were approved by the CWRU Institutional Animal Care and Use Committee (IACUC) in accordance with approved IACUC protocols (2019-0065). Mice were housed in standard microisolator cages and maintained on a defined, irradiated diet and autoclaved water. The experimental procedures used in this arm of animal experiments were based on existing dosage and treatment regimens used to treat felines suffering from FIP. Mice were treated with 3 mg/kg PI once daily by intraperitoneal (IP) injection for 3 consecutive days. After the final PI treatment (day 3), the mice were euthanized 8 h after the final treatment. All animals were observed daily for signs of illness.

SASS & SASS Animals: Husbandry and experimental procedures were approved by the Company’s Institutional Animal Care and Use Committee (IACUC). Eight females that were over one year old, CD-1 retired breeders (Charles Rivers lab, Garfield Heights, OH, USA) and previously used for a toxicity study of PI were administered with 3 mg/Kg of PI.

Complete Blood Count Analysis: Peripheral blood was collected into Microtainer EDTA tubes (Becton-Dickinson, Franklin Lakes, NJ, USA) by submandibular cheek puncture. Blood counts were analyzed using a Hemavet 950 FS hematology analyzer (Drew Scientific, Plantation, FL, USA).

Quantification of bone marrow (BM) and splenic immune cell populations: Bone marrow cells were obtained by flushing hindlimb bones, and splenocytes were obtained by mincing spleens. Cells were stained with antibodies against CD45R/B220 (RA3-6B2), CD11b (M1/70), CD3e (500A2), B220(RA3-6B2), CD4 (RM4-5), CD8 (53-6.7), Ly-6G and Ly6C (RB6-8C5), TER-119 (TER-119), Ly-6A/E (D7), CD117 (2B8), F4/80 (Cl:A3-1) and CD61 (2C9.G2), and data was acquired on an LSRII flow cytometer (BD Biosciences, Franklin Lakes, NJ, USA). Analysis was performed on FlowJo software v10.10 (Ashland, OR, USA).

### 2.4. Metabolomic Studies Using Mass Spectroscopy

Internal Standard (IS) solution preparation: The working stock solution of PGE2-d4 at 10.0 μg/mL was prepared by diluting 20.0 μL of the 500 μg/mL with 980 μL of diluting solvent methanol/acetonitrile/water (2:2:1).

Cell sample preparation: Metabolomic studies with THP-1 were performed using three different treatment regimens corresponding to untreated cells, treatment with n-butanol (vehicle), and treatment with 75 μg/mL PI for 24 h, the same condition used in the THP-1 flow cytometry study. After 24 h, cells were harvested by centrifugation and the resulting cell pellet was suspended in 1.0 mL deionized water and sonicated for 30 s to lyse the cells. The protein concentration was measured using the Pierce BCA protein assay kit (Thermo Fischer Scientific) to normalize the cell growth factors across different conditions. The final protein concentration was adjusted to 100 μg/mL by adding an appropriate volume of deionized water. The metabolites of the cell samples were extracted using liquid–liquid extraction by modifying the Folch method. In detail, 1.00 mL of each cell lysate of every experimental condition was added with a 3.00 mL extraction solvent of chloroform/methanol (1/1), and the mixture was vortexed vigorously for 2.00 min and placed in an ice bath for 15.0 min. After the phase separation, the mixture was centrifuged at 2000× *g* at 4 °C for 15.0 min, and the lower organic layer was carefully aspirated without disturbing the protein disc in the center and collected in a fresh culture tube. The remaining aqueous phase and protein disc were added with 3.00 mL chloroform/methanol (2/1), and the mixture was vortexed for 2.00 min and incubated in an ice bath for 15.0 min. Again, the mixture was centrifuged at 2000× *g* at 4 °C for 15.0 min, and the lower organic phase was aspirated and mixed with the previous aspirated aqueous phase and mixed. This organic layer was evaporated under a nitrogen stream using the N-EVAP TM 111 nitrogen evaporator from Organomation (West Berlin, MA, USA). Once the samples were dried, the internal standard solution and reconstitution solvent (acetonitrile/methanol/water with a ratio of 2:1:1) were added, making the final internal standard concentration at 2.00 μg/mL.

Plasma samples prepared from the whole blood of mice: The plasma samples under three experimental conditions, namely untreated, vehicle (treated with placebo), and treated (treated with PI), were subjected to protein precipitation for the extraction of metabolites. An extraction solvent acetonitrile/methanol (2:1) volume of 960 μL was added to 240 μL of plasma. The mixture was vigorously vortexed for 2.00 min and incubated overnight at −20 °C to enhance the precipitation of proteins. The following day, the samples were centrifuged at 13,000× *g* for 15.0 min at 4 °C, and the supernatant was collected in fresh borosilicate glass culture tubes. The supernatant was dried under a stream of nitrogen and reconstituted with reconstitution solvent (acetonitrile/methanol/water with a ratio of 2:1:1) and internal standard solution (IS, see above for preparation method, final concentration 2.00 μg/mL).

UHPLC-QTOF/MS analysis: The data were acquired on Agilent’s Infinity II 1290 liquid chromatography unit with a binary pump, a degasser, a multisampler, and a column oven compartment coupled with a 6545-quadrupole time-of-flight mass spectrometer. The data for cell and plasma samples were acquired in both positive and negative electrospray ionization (ESI) modes, using a mobile phase pair containing solvent A, which comprised 5 mM of ammonium acetate aqueous solution with 0.1% acetic acid, and solvent B, which comprised acetonitrile/methanol (80:20) with 5 mM of ammonium acetate and 0.1% acetic acid with a gradient elution profile as follows: 0.00–1.00 min (40% B), 12.0 min (75% B), 20.0 min (85% B), 28.0–38.0 min (100% B), 40.0 min (75% B), and returning to 40.0–45.0 min (40% B). Each chromatographic run included a 10 min column pre-equilibration at initial conditions (40% B). The chromatographic separation was carried out using a Waters XSelect HSS T3 (2.1 × 150 mm, 2.5 μm) analytical column (Milford, MA, USA) at 35 °C with a sample volume of 5 μL. Each biological sample was injected four times as technical replicates in both ESI modes.

Data processing: The data for the cell and plasma samples obtained under three experimental conditions (control, vehicle, and treated) were acquired using Agilent MassHunter Data Acquisition software (Version: B.10.1.48) in both ESI modes. The raw data (.d) files were imported to Agilent MassHunter Qualitative Analysis software (Version: B.10.0.1) to assess the chromatographic peak shapes, retention time profiles, and mass spectral background noise. The retention time across all data files under each experimental condition for the cell and plasma samples under each ESI mode was corrected using internal standard solution analytical runs with a minimum spectral height. This ensured the correction of retention time deviations across different analytical runs and the removal of background noise in the mass spectrum. The batch recursive molecular feature extraction strategy was used to identify molecular features. The specific parameters and values are as follows: the extraction parameters included a minimum mass spectral peak height of 1500 counts, allowed ion species of [M+H]^+^, [M+Na]^+^, and [M+NH_4_]^+^ for the positive ion mode, and [M−H]^−^ for the negative ion mode, the isotope model of common organic molecules without halogens, and the limit assigned charge states to a range of 1–2; the compound filters were set by default; the compound binning and alignment parameters included a retention time tolerance of 0.10% + 0.30 min and a mass tolerance of 20.00 ppm +2.00 mDa; and the post-processing filters were set at an absolute height of at least 5000 counts for mass spectral peaks, a molecular feature extraction score of at least 75, and a minimum match of molecular feature at 75% (this meant a molecular feature must be present in 3 out of 4 replicate runs in each experimental condition to be included). For the find by ion, the matching tolerance and scoring parameters included a mass score at 100, isotope abundance and spacing scores at 60 and 50, respectively, and a retention score of 0; the EIC peak integration and filtering parameters included an absolute height of at least 7000 counts for chromatographic peaks; the spectrum extraction and centroiding parameters were set by default; and the post-processing filters included an absolute height of at least 7000 counts for chromatographic peak heights and a target score of at least 75.00. This was performed by uploading data files of different experimental conditions (control, vehicle, and treated) for cell samples obtained in positive ESI data acquisition mode and separately for those obtained in negative ESI data acquisition mode. Similar was performed for plasma samples. Finally, the data of all experimental groups by each ionization mode obtained from the operations of “molecular feature extraction” and “find by ion” were exported as profinder archive (.pfa) files from the Agilent MassHunter Profinder software (Version: B.10.0.1).

Multivariate analysis: The reproducibility of the UHPLC-QTOF/MS data was assessed using multivariate principal component analysis (PCA) using MetaboAnalyst V6.0. In detail, using the m/z, retention, and peak area data of all molecular features identified by profinder software, (Version: B.10.0.1) different PCA plots were generated. These data were exported in separate .csv files for every experimental condition (i.e., control, vehicle, and treated) in both ESI data acquisition modes (positive and negative) for the samples (cell and plasma). These replicates of the individual condition .csv files were grouped in a folder and zipped together as .zip files (four files, positive ESI mode for cell samples, negative ESI mode for cell samples, positive ESI mode for plasma samples, and negative ESI mode for plasma samples). These individual .zip files were uploaded to MetaboAnalyst under the statistical section as MS (mass spectrum) peak list data. The retention time and mass tolerance were set to 30.0 s and 0.025 Da. Data filtering was performed using an “interquartile range” (IQR) of 5% to remove variables and increase accuracy. Further, the data were normalized using the “internal standard” molecular feature, and the data were log-transformed to the base 10 and auto-scaled. Then, they were submitted for multivariate analysis to generate 2D PCA plots.

Metabolite identification and statistical analysis: The (.pfa) files containing molecular feature data were uploaded to Agilent Mass Profiler Professional (MPP) software (Version: B.15.1.2) for statistical analysis and metabolite identification. This was performed separately for the positive and negative data acquisition modes for cell samples, and similarly for plasma samples. The metabolomics data uploaded to MPP were normalized using the internal standard (PGE2-d4) spiked in each sample to correct the signal fluctuations between the analytical runs. The data for vehicle and treated cells were adjusted to the median values of the control data to assess the regulation of metabolites under different experimental conditions. The molecular features were annotated using Agilent’s METLIN accurate mass lipids and metabolites libraries with a passing score of 75.0. One-way ANOVA statistical analysis followed by Tukey’s HSD test was applied to identify statistically significant metabolites (*p*-value ≤ 0.05) with Benjamini–Hochberg correction for FDR. The log2 values of peak areas of the metabolites were used to identify the significantly regulated metabolites between pairs of experimental conditions such as control vs. vehicle, control vs. treated, and vehicle vs. treated. Metabolites with a log2-fold change greater than 2.0 (or a peak area greater than 4-fold) were considered to be significantly regulated metabolites between the pair of noted experimental conditions.

**Figure 2:**
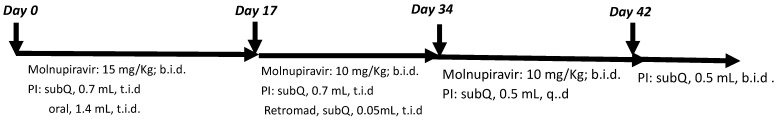
Molnupiravir/PI combo treatment regime for a FeLV-positive cat with wet FIP.

Treatment protocol for a feline leukemia positive/effusive FIP-positive cat with molnupiravir and PI: Buddy Boy was a stray domestic short-haired male cat who tested positive for feline leukemia with an IDEXX SNAP combo test during neutering surgery. He was transferred into foster care and later developed a distended abdomen within a month. His blood count and chemistry profiles were obtained using the ProCyte Dx Hematology Analyzer and the Catalyst One Chemistry Analyzer (IDEXX, Westbrook, ME, USA). The abdominal effusion was subjected to PCR testing for feline coronavirus conducted by IDEXX. Buddy Boy was then placed on oral molnupiravir and PI using the protocol summarized in Figure 2.

### 2.5. Statistical Analysis

For the cell culture and blood samples flow cytometry data, all values were tabulated graphically with error bars corresponding to the standard error of the mean. Analysis was performed using GraphPad Prism software 10.4.2. Unless otherwise noted, an unpaired two-tailed Student’s t-test was used to compare treatment groups. The standard deviation measures varied within each mouse in different treatment groups. A type 1 error is measured by the significance level, which is usually fixed at the level of 5% (*p* = 0.05). Statistical tests using Student’s t-test or the Chi-square test or more complex tests such as ANOVA were performed using computer software available on programs such as Kaleidagraph v5, which are routinely used for graphical analyses.

For the metabolomic data, one-way ANOVA statistical analysis followed by Tukey’s HSD test was applied to identify statistically significant metabolites (*p*-value; ≤0.05) with Benjamini–Hochberg correction for FDR. The log2 values of peak areas of the metabolites were used to identify the significantly regulated metabolites between pairs of experimental conditions such as control vs. vehicle, control vs. treated, and vehicle vs. treated. Metabolites with a log2-fold change greater than 2.0 (or a peak area greater than 4-fold) were considered to be significantly regulated metabolites between the pair of noted experimental conditions.

## 3. Results

**Figure 3:**
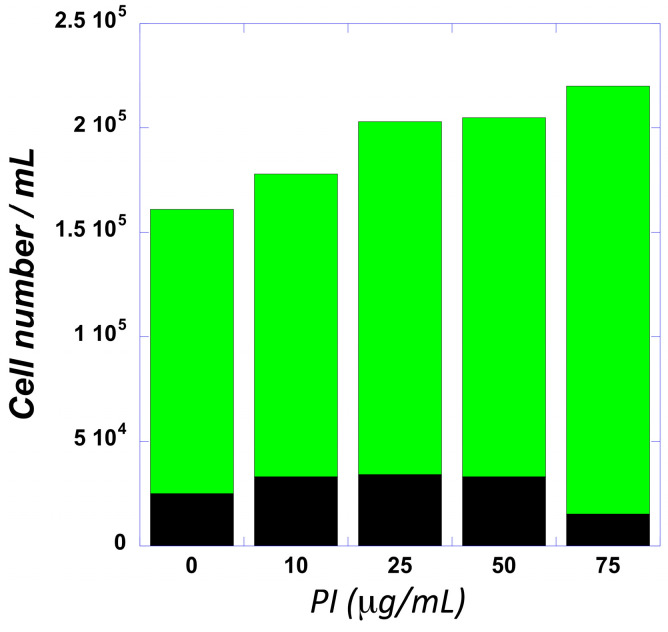
The dose–response curve for PI plotting viable (green) and non-viable (black) THP-1 cells as a function of increasing PI concentrations.

Metabolomic studies. The biochemical effects of PI were quantified by comparing the metabolomic profiles of THP-1 cells treated with PI (ex vivo) with whole blood from mice treated with equivalent concentrations of PI (in vivo). In both ex vivo and in vivo studies, PI-treated samples were compared against n-butanol as the vehicle control. The first study employed THP-1 cells, which are an established cell model used to study inflammation and differentiation into macrophages [41]. The goal here was to first examine the potential effects of acute PI treatment (24 h) on the viability and proliferation of THP-1 cells. Figure 3 provides a dose–response curve examining cellular proliferation as a function of increasing PI concentrations (0–75 μg/mL) over a 24 h time period. These data show a steady increase in the total cell number as a function of an increasing PI concentration. The highest dose tested (75 μg/mL) produced a 40% increase in total cell number. Since there was no statistical difference in the number of non-viable cells (black bars) treated with 75 μg/mL PI, the higher cell number results from an increase in viable cells (green bars) compared to cells treated with n-butanol (vehicle). This result was confirmed using Annexin V/Propidium Iodide uptake to examine early and late-stage apoptosis. Nearly identical levels of early and late-stage apoptosis in THP-1 cells treated with n-butanol versus 75 μg/mL PI were detected. Based on these data, the metabolic profiles of THP-1 cells were measured by comparing treatment with 75 μg/mL PI to treatment with n-butanol.

We next examined the effects of acute PI treatment in mice. In this study, mice received 3 mg/kg PI via intraperitoneal injection on a Q.D. schedule and were then euthanized 8 h after the third and final dose. Mice treated with the vehicle, n-butanol, and no treatment were included as controls. All mice were housed in micro-isolators and thus not exposed to or challenged with any pathogens. Thus, the metabolomics profile evaluates the effects of PI on non-infected animals.

**Figure 4:**
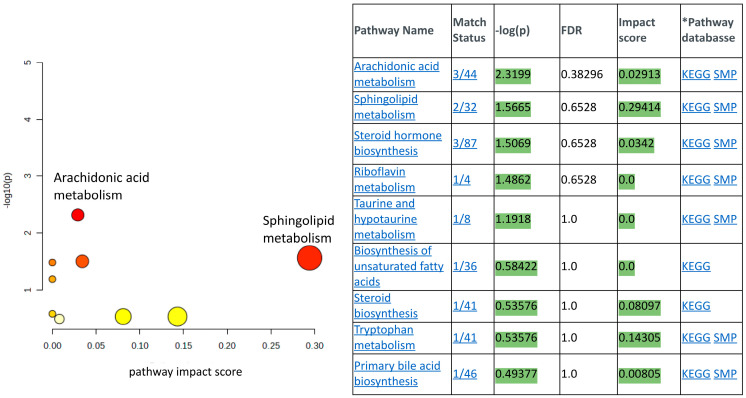
The pathway analysis results of the significantly regulated metabolites identified. The y-axis denotes −log10(p) values representing the statistical significance of the identified metabolites participating in the pathway (generated from one-way ANOVA), while the x-axis represents the pathway impact score (calculated as sum of centrality of identified metabolites normalized by the sum of the centralities of all metabolites in the pathway). The color and size of the nodes (yellow to red, small to large) shows the significance levels of the metabolites in the pathway, with red being the most significant. The KEGG pathway database and the SMP, small molecule pathway database, were used as the reference pathways.

Supplementary Figure S1 provides representative LC-MS chromatograms of the metabolites in the THP-1 cell lysates. The Agilent MassHunter Profinder software (Version: B.10.0.1) was used for time alignment and signal normalization by exogenous internal standard and molecular feature extraction (batch recursive feature extraction for small molecules). The MPP program was used to achieve internal standard and protein normalization and perform the statistical analyses, filtered by frequency and background subtraction. The same strategy was used to obtain the metabolomic profiles of mice treated with n-butanol, PI and no treatment; the LC-MS chromatogram data are not shown. Cross-referencing sample sets from whole blood versus THP-1 cell lysate sample sets using the statistical analysis procedure described in the method section above identified nine common metabolites that can be categorized in the pathway analysis table shown in Figure 4. A list of all the common metabolites found in THP-1 cell lysates and mice blood samples whose levels were perturbed by the treatment of PI over vehicle and/or no PI treatment is supplied in the Supplementary Data Section. Figure 4 was generated using the pathway analysis approach reported by Liu et al. [42]. In brief, metabolic pathway enrichment analysis and topology analysis were conducted through pair-wise comparisons of the global metabolomic profiles of the mice blood samples and THP-1 cell lysates treated with n-butanol, the vehicle, and with and without PI, as described in the method section. The -log *p* values, which were computed by the software stated in the mass spectrometry data analysis section, measure the statistical significance of the metabolites whose levels were altered in response to one of the treatments (vehicle, no PI and with PI) in the pathways listed in Figure 4. The pathway impact score on the *x*-axis measures the level of representation of the detected metabolites in the specific metabolic pathway. In this metabolomic study, we identified that sphinganine and ceramide were the common metabolites upregulated by PI treatment. Sphinganine and ceramide are two out of the thirty-two metabolites used to define the sphingolipid metabolic pathway (see match status in Figure 4). The pathway impact scores attributed to these two metabolites were computationally calculated as the sum of the centrality of identified metabolites normalized by the sum of the centralities of all metabolites in the pathway, yielding a value of 0.29. Plotting the impact score versus the -log *p* value allows for the visualization of the relative impact of PI on sphingolipid metabolism compared to the other eight pathways that had their metabolite levels perturbed by PI treatment.

**Figure 5:**
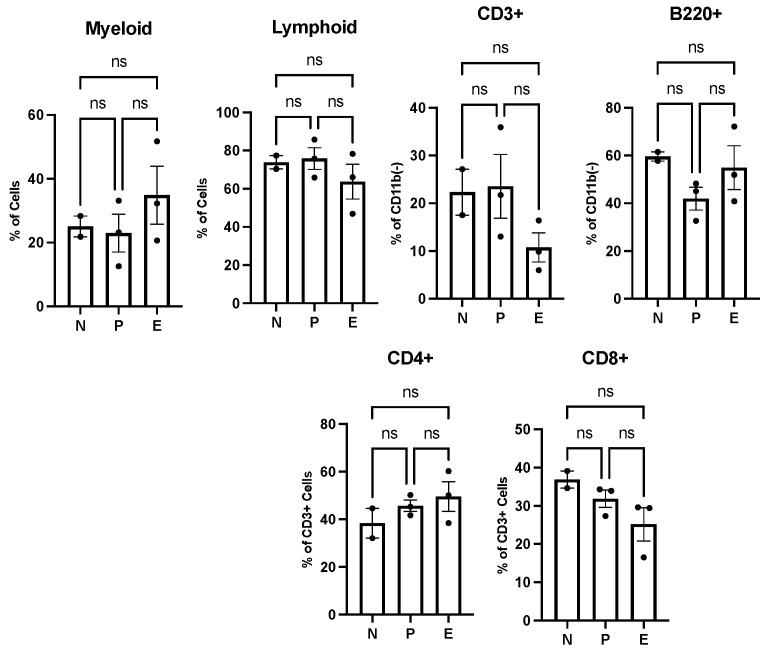
Polyprenyl immunostimulant (PI) moderately impacts the frequencies of B and T cells in peripheral blood. In this experiment, N denotes two untreated mice; P denotes three n-butanol-treated mice and E denotes three PI-treated mice. All mice were treated and harvested as described in the Methods section. Blood count and immune cell populations were determined as described in the Methods section. Statistical analyses were performed using GraphPad Prism software version 10.4.2 as described in Section 2.5; ns means not significant.

We performed immunophenotypic analyses using flow cytometry to quantify whole blood, bone marrow and splenic immune cell populations. The metabolomic studies of whole blood samples obtained from healthy mice indicated the upregulation of sphinganine and N-acylsphingoine, also known as ceramide metabolism, both of which are precursors of sphingosine-1-phosphate (S1P). Ceramide and S1P are signaling molecules that modulate the immune cell population and regulate T-cell-mediated innate immunity [43]. Sphingosine-1-phosphate (S1P) can mediate the trafficking of T and B cells, whereas ceramide can signal the activation and proliferation of T cells. As such, we expected to detect a difference in the immune cell population in mice treated with PI versus without PI. To this end, immunophenotyping flow cytometry analyses were performed using whole blood samples harvested from the same mice used in the metabolomic studies. Figure 5 summarizes the flow cytometry data examining myeloid and lymphoid cells under various treatment conditions. In general, a slight increase in myeloid (B cells) cells with a concomitant decrease in lymphoid cells (T cells) was observed with PI treatment. While myeloid and lymphoid cells both originate in bone marrow, they have different biological functions. Myeloid cells are part of the innate immune system and can produce other immunological cells including neutrophils, monocytes, macrophages, and red blood cells. While the levels of myeloid cells were slightly elevated after PI treatment, there was no significant change in B220+ cells. B220 is a tyrosine phosphatase associated with cell differentiation and signaling, and is commonly used as a pan-B cell marker. Lymphoid cells represent key components of the adaptive immune system and ultimately develop into B lymphocytes and T lymphocytes. The analyses here show that amongst lymphoid cells, the levels of CD3+ and CD8+ cells decreased while CD4+ cells increased after PI treatment. The change in CD4+ and CD8+ is noteworthy since a high CD4/CD8 ratio can be caused by various factors, most notably acute viral infections. Since mice were not exposed to any viral pathogen, the higher ratio caused by PI treatment suggests that PI “primes” the immune response to combat viral infections.

**Figure 6:**
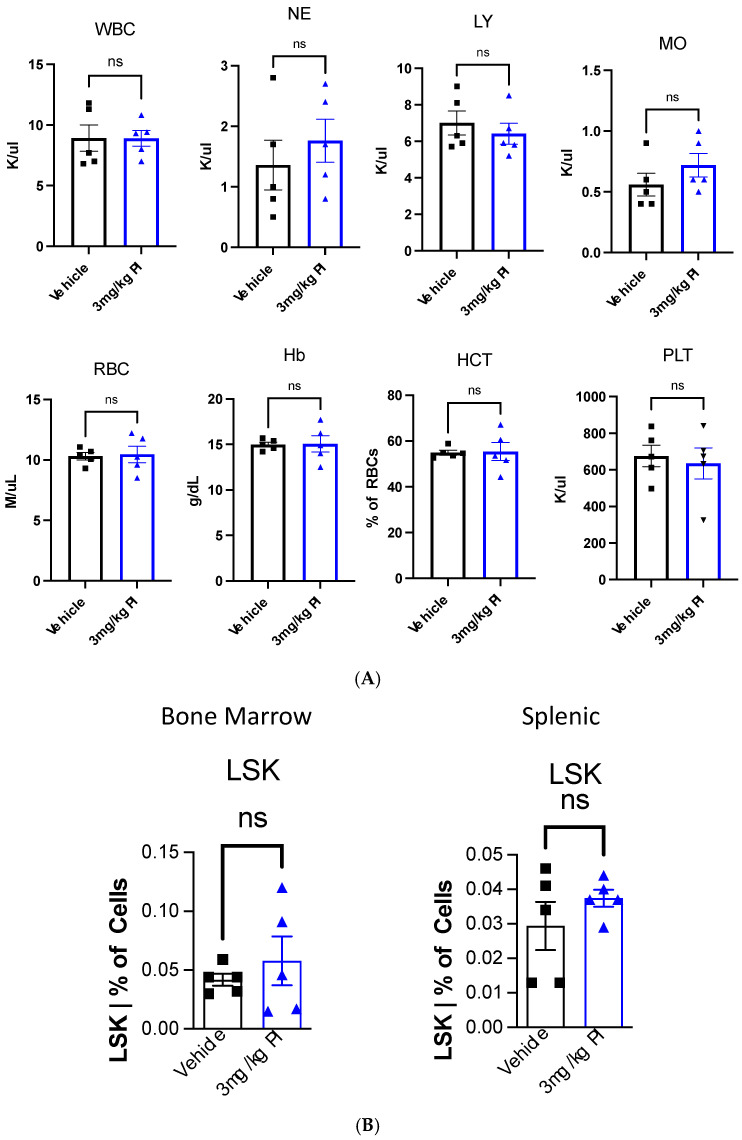

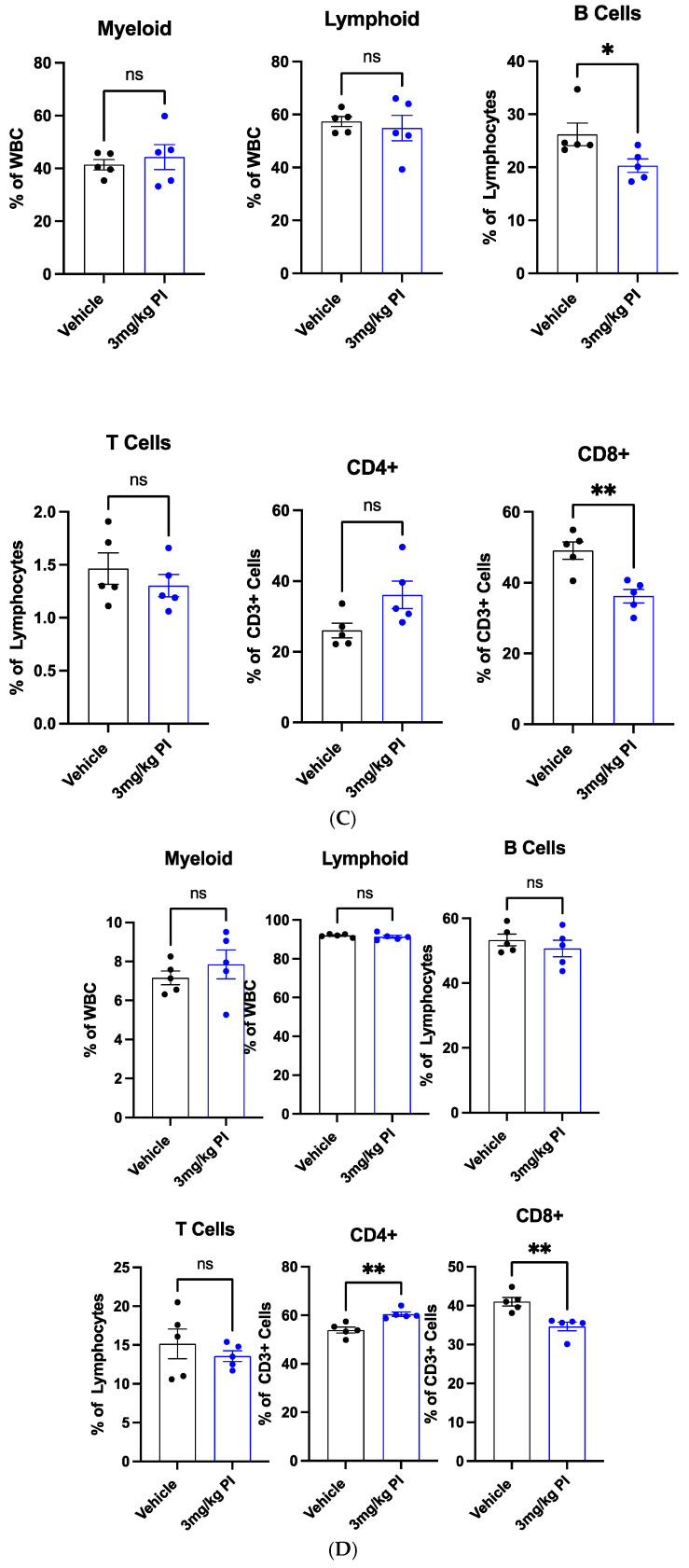
(**A**) Polyprenyl immunostimulant (PI) does not impact the composition of peripheral blood. In this experiment, five mice were used in each arm. All mice were treated and harvested as described in the Methods sections. The blood count and immune cell populations were determined as described in the Methods section. Statistical analyses were performed using GraphPad Prism software version 10.4.2 as described in Section 2.5; ns means not significant. (**B**) PI does not significantly alter the BM and splenic HSPC frequencies. In this experiment, five mice were used in each arm. All mice were treated and harvested as described in the Methods sections. Immune cell populations were determined as described in the Methods section. Statistical analyses were performed using GraphPad Prism software version 10.4.2 as described in Section 2.5; ns means not significant. The raw flow cytometry data used to generate this plot are supplied in the Supplementary Data Section. (**C**) PI reduces BM B cells and alters BM T cell frequencies. In this experiment, five mice were used in each arm. All mice were treated and harvested as described in the Methods section. Immune cell populations were determined as described in the Methods section. Statistical analyses were performed using GraphPad Prism software version 10.4.2 as described in Section 2.5; ns means not significant; * refers to *p* ≤ 0.05 whereas ** refers to *p* ≤ 0.01. The raw flow cytometry data used to generate this plot are supplied in the Supplementary Data Section. (**D**). PI alters the splenic T cell but not B cell frequencies. In this experiment, five mice were used in each arm. All mice were treated and harvested as described in the Methods section. Immune cell populations were determined as described in the Methods section. Statistical analyses were performed using GraphPad Prism software version 10.4.2 as described in Section 2.5; ns means not significant; ** refers to *p* ≤ 0.01. The raw flow cytometry data used to generate this plot are supplied in the Supplementary Data Section.

Figure 3 shows that PI induces the proliferation of THP-1 cells. THP-1 monocytes can be induced by phorbol 12-myristate-13-acetate (PMA) to differentiate into macrophages [44]. Given this consideration, we decided to examine the effect of PI the hematopoietic stem and progenitor cells (HSPCs) and the differentiated blood cells. To this end, ten 9-week-old female C57/Bl6 mice that had never been exposed to PI were treated with the same protocol as the aged mice used in the metabolomic study. In addition to sampling the effect of PI on mature blood cells, we evaluated the effect of PI on HSPCs by monitoring the number of LSK cells in the bone marrow and the spleen. LSK cells are negative linkage cells that express the cell surface protein Stem cell antigen 1 (Sca-1) and tyrosine kinase receptor c-Kit, also known as CD117. Peripheral blood, bone marrow, and splenic tissue were harvested from mice, and the cells were stained with fluorescently labeled antibodies for cell surface immunomarkers specific to the cell types; this was followed by flow cytometry imaging. Figure 6A shows that the cellular composition of peripheral blood is comparable between PI and the vehicle n-butanol treatment. For example, the levels of white blood cells (WBC), red blood cells (RBC), hemoglobin (Hb), and platelets (PLT) remain invariant after PI treatment compared to the vehicle-treated mice. These data indicate that PI does not affect this parameter. Figure 6B shows the flow cytometry results of the bone marrow versus the splenic HSPCs, represented by the LSK cells; PI does not significantly impact the cell populations. Figure 6C shows that PI decreases the percentage of B cells and CD8+ cells in BM, but increases CD4+ cells. This results in a high CD4+/CD8+ ratio, which was also observed in studies examining the effects of PI in peripheral blood samples. Figure 6D shows that PI affects the population of splenic T- but not B-cells. Surprisingly, PI treatment causes a decrease in splenic CD8+ cells but does not alter the levels of splenic CD4+ cells. Regardless, the net effect still results in an overall higher CD4+/CD8+ ratio. Collectively, the data presented in Figure 6 show that PI affects only differentiated blood cells.

A case study on using PI to enhance the therapeutic property of molnupiravir was performed. In light of the whole blood and THP-1 cell studies, along with published data showing that PI modulates the innate immunity of animals [45], we conducted a proof of principle study to evaluate the ability of PI to enhance the therapeutic potential of the antiviral drug molnupiravir. In humans, molnupiravir has shown efficacy in alleviating mild symptoms of COVID-19. In felines, molnupiravir has been used successfully as an oral medication to treat FIP in non-immune-compromised hosts [31,46,47]. However, no study on the efficacy of molnupiravir in treating immune-compromised hosts has been found. For a proof-of-principle study, we treated a stray feline that was displaying the effusive form of FIP and was confirmed positive for the progressive stage of feline leukemia viral infection with a combination of PI and molnupiravir. Since FIP is fatal when left untreated, we did not include a control for comparison for ethical reasons. Furthermore, the subject of interest here was found to be a 2-year-old stray with an unknown history. Finding a comparable feline to serve as a control would be impossible. To assess the effect of PI, we compared the treatment duration needed to bring the feline to remission with the molnupiravir treatment duration used in the literature to treat unimmune-compromised cats [31,46,47]. Using the protocol outlined in Figure 2, we monitored the feline’s FeLV status using the IDEXX FeLv Quant real PCR, the FIP status, and the progression of both diseases according to the feline’s complete blood count (CBC) and chemistry profile. The IDEXX FeLV Quant real PCR test value obtained on day 17 and day 31 of the molnupiravir/PI combo treatment was 14.19 × 10^6^ copies/mL and 41.97 × 10^6^ copies/mL, respectively. The quant real PCR value for FeLV did not change significantly during the course of treatment. Table 1 shows the blood count and chemistry profile values that changed during the course of treatment. There was a drop in the hematocrit (HCT) value after the administration of PI/molnupiravir. Non-regenerative anemia is often a phenotype of FeLV or FIP infection. Since the subject was already receiving PI and molnupiravir to treat FIP, we attributed the drop in HCT to a manifestation of FeLV infection exacerbated by FIP. As such, we added a low dosage of the antiviral agent RetroMad 1 to control the FeLV flareup [48]. After 35 days of treatment, the CBC and chemistry profiles of the subject returned to normal ranges. The abdominal exudate caused by the effusive form of FIP was eliminated, but the progressive stage of FeLV infection persisted. The five-week treatment time needed to attain remission in FIP is less than the reported 12-week (84 days) protocol for non-FeLV subjects.

## 4. Discussion

Polyprenyl immunostimulant (PI) is an immune modulator that is effective in treating non-effusive FIP and FRV [32,33,34,35]. The immunomodulating activity of PI was detected by the increase in the mRNA levels of the pro-inflammatory cytokines IL1-β, IL-8 and THFα, along with an increase in the expression of CD11b in monocytes isolated from the whole blood of felines [38]. The efficacy of PI in treating viral infection has been evaluated through monitoring the clinical signs, total blood counts and chemistry profiles of the treated felines. As such, there are no experimental procedures available to identify the cellular and molecular targets. To fill the noted deficiency, this study provides the first quantitative pilot study identifying the biochemical and/or biomarkers of PI that can be used to track its performance in relation to immune functions. This proof-of-concept study utilized metabolomics to identify two metabolites, sphinganine and ceramide, which are upregulated by PI in healthy mice and in THP-1 cells. Both metabolites are key intermediates in the sphingolipid metabolic pathway and play significant roles in modulating innate immunity in mammals. Sphingolipids are known to be associated with the activation and/or regulation of cell-mediated immunity [43]. Since PI structurally resembles sphingolipids due to the presence of phosphate moieties at the end of its hydrophobic carbon chain, it is possible that PI interacts with one or more proteins in the sphingolipid metabolic pathway to initiate an immune response.

Sphinganine and ceramide are metabolites that lead to the production of sphingosine-1-phosphate (S1P) [49]. Ceramide and S1P are signaling molecules that mediate the trafficking of T and B cells. Additionally, ceramide serves as a signal to modulate the functions of T cells [50]. Performing the immunophenotypic analyses via fluorescent flow cytometry allowed us to assess PI’s impact on different blood cell distributions, especially on CD4+ and CD8+ T cells. The data presented in Figure 6 corroborate the effects of PI on the upregulation of the sphingolipid metabolism, leading to changes in the T cell subset distribution. There are increased CD4+ and decreased CD8+ cells within the T cell populations; hence, there is an overall increase in the CD4/CD8 ratio in the cell profile. In human immunodeficiency, an increased CD4/CD8 ratio in CBC is considered an improvement in immunity [51]. The detection of an increase in CD4/CD8 in the blood of PI-treated healthy mice further proves the immunotherapeutic potential of this compound. When interpreted along with the metabolomic data and the steady-state hematopoiesis study described in the Results section, this flow cytometry result suggests the impact of PI on T-cell-mediated immunity.

Since the mice and THP-1 cells used in this study were treated under pathogen-free conditions, the quantitative nature of metabolomic and flow cytometry allows the setting of reference standards in the disease-free samples such that the effect of PI in combating viral infection or other hematopoietic injury can be quantitatively assessed. But the metabolomic experiments performed in this study were monitored at a specific concentration of PI at only one time. As such, additional metabolomic experiments conducted at different PI treatment times and concentrations will be needed to provide a more robust analysis, which will provide more support for the participation of PI in sphingolipid metabolism, and reveal the molecular targets. The findings of this study demonstrate the technical and conceptual feasibility of combining metabolomic and immunophenotyping to analyze an immune cell culture model (THP-1) and animal blood samples to quantitatively monitor the immunomodulating properties of PI, and identify a lead target pathway for PI. To further demonstrate the immunomodulating properties of PI in managing viral infection in mammalian hosts, we performed a proof-of-principle case study to demonstrate that adding PI to molnupiravir therapy reduces the treatment duration in immunocompromised subjects. Collectively, the findings generated from this study establish the basis and approaches for studying the molecular mechanism of PI and evaluate the ability of PI to synergize the therapeutic efficacies of other antiviral drugs in general.

In summary, PI has been successfully used as an immunomodulator to combat viral infections in felines. However, its impact on healthy animals has not been evaluated until now. The discovery of metabolites linked to the sphinganine and ceramide metabolism in PI-treated mice indicates the upregulation of innate immunity, which may explain its immunotherapeutic properties. Applying mice as a translational model will significantly facilitate the identification of the molecular targets of PI, especially in inflammation and age-related diseases. Genetic knock-down and knock-out mice are available for conducting a comparable metabolomic study, and data can be quantified and compared with the findings of this study.

## 5. Conclusions

PI is the only immunomodulator that shows efficacy in treating herpes and FIP in felines [32,35]. Because FIP is a coronavirus infection and the symptoms of FIP are similar to those of a human coronavirus infection, such as COVID-19 [52], the therapeutic benefit of PI could potentially be exploited to treat human viral infections. Currently, the cellular and molecular targets of PI are not known, and quantitative analytical methods to compare the effectiveness of PI in animals and cell culture models are not available. This work was therefore performed as a pilot study to assess the feasibility of using a combined metabolomic/immunophenotypic flow cytometry approach to identify quantifiable immunological parameters in THP-1 cells and in mice to determine the efficacy of PI. Metabolomic studies of THP-cell lysates and the whole blood of mice identified ceramide and sphinganine as the immunochemical markers for monitoring the action of PI in un-insulted cells and mice. Ceramide and sphinganine both participate in the signaling of T cell activation, proliferation, and distribution [43]. The detection of a change in the CD4+/CD8+ cell ratio but the neglectable effect on B cells in the blood and lymphoid organs, bone marrow, and spleen indicate that PI modulates the functions of T cells through the upregulation of the sphingolipid pathway. The immunotherapeutic benefit of PI as a synergetic agent to combat viral infection was demonstrated by the shortened molnupiravir treatment duration in an immunocompromised feline co-infected with FeLV and FIP viruses. This study provided an experimental approach to measuring the efficacy of PI in cell cultures and blood samples, revealed a plausible molecular pathway targeted by PI in vivo, and demonstrated the benefit of PI as an immunotherapeutic agent in animals.

## Figures and Tables

**Table 1:** Data were generated by the ProCyte Dx Hematology Analyzer and the Catalyst One Chemistry Analyzer. Abnormal values are highlighted in red. Treatment ended on day 31.

	Day 0	Day 17	Day 31	Day 56	Normal Range
Hematocrit (%)	26.7	22.5	33.6	41.2	30.3–52.3
Neutrophils (K/mL)	10.61	6.12	9.37	3.65	2.3–10.29
Eosinophils (K/mL)	0	0.17	0.63	0.22	0.17–1.57
BUN (mg/dL)	12	20	21	23	16–36
total protein (g/dL)	7.0	9.5	7.7	7.2	5.7–8.9
Globulin (g/dL)	4.8	6.4	5.0	3.9	2.8–5.1
ALP (U/L)	<10	54	43	103	14–111
Total bilirubin (mg/dL)	1.5	0.7	0.3	<0.1	0–0.9

## Data Availability

The original contributions presented in this study are included in the article/Supplementary Material. Further inquiries can be directed to the corresponding author(s).

## Supplementary Materials

The following supporting information can be downloaded at: https://www.mdpi.com/article/10.3390/cells14100752/s1, Figure S1: Chromatograms of the metabolites detected by LC-QTOF/MS 6545 in THP-1 cells; Figure S2: Flow Cytometry data of immunophenotyping whole blood, bone marrow and splenic immune cells; Table S1: Common metabolites that differ by 2-fold, found in PI treated THP-1 and mice blood cells. Metabolites involved in the sphingolipid metabolism are highlighted in yellow.
