# Supplementary material for: Utilization of Flow Cytometry, Metabolomic Analyses and a Feline Infectious Peritonitis Case Study to Evaluate the Physiological Impact of Polyprenyl Immunostimulant
Source: Cells. 2025 May 21;14(10):752. doi: 10.3390/cells14100752 (PMC12110054; doi:10.3390/cells14100752)
Supplement: Supplementary file 1 [file cells-14-00752-s001.zip › cells-3590469-Supplementary Figure.pdf]

Supplementary Data

Figure S1. Chromatograms of the metabolites detected by LC-QTOF/MS 6545 in THP-1 cells.

Metabolites recovered in the aqueous layer of the THP-1 cell extraction process under negative ion detection mode. Control: untreated cell; PI: cells treated with PI

Neg_Aq

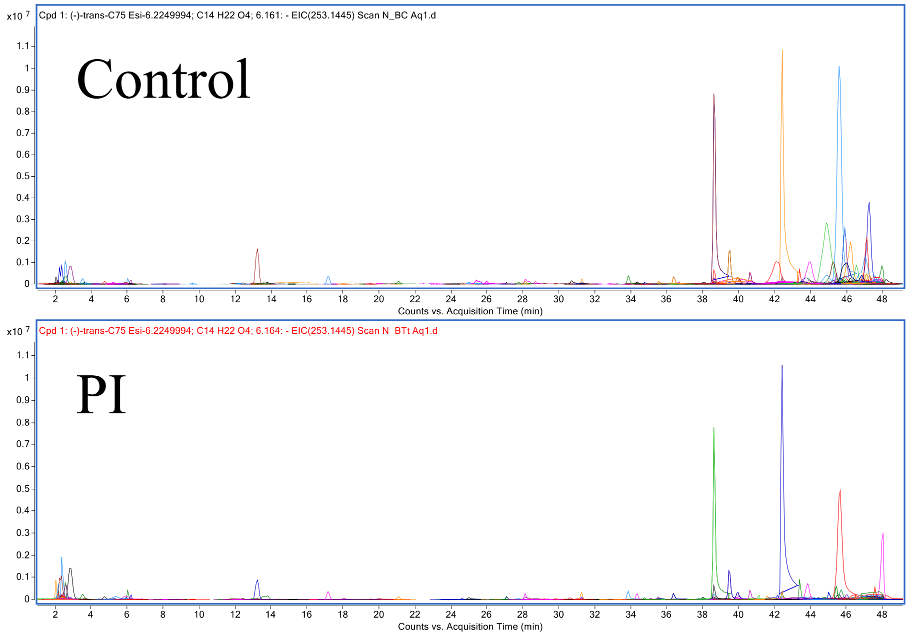

Metabolites recovered in the aqueous layer of the THP-1 cell extraction process under negative ion detection mode. Butanol: cells treated with n-butanol, the vehicle; PI: cells treated with PI

Neg_Aq

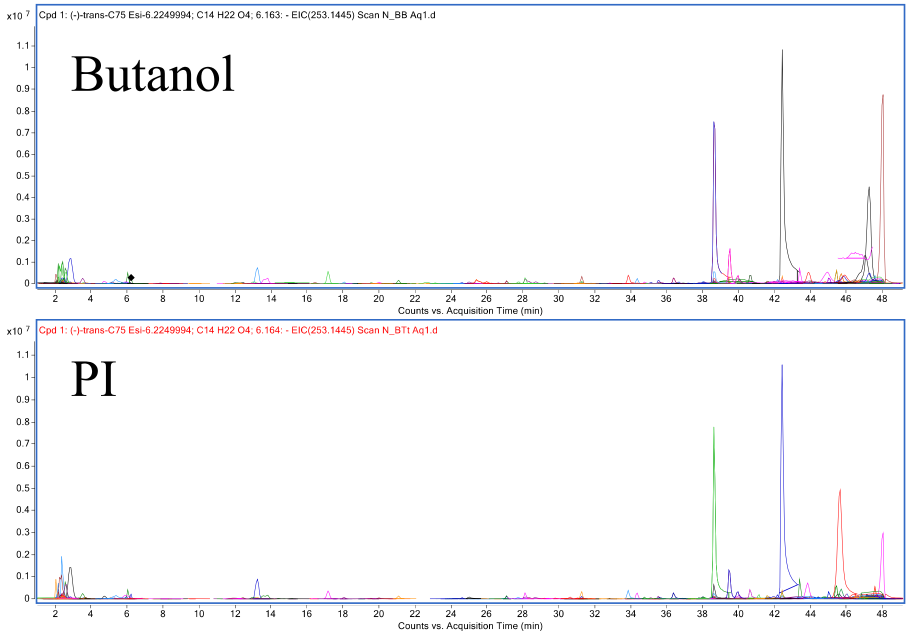

Metabolites recovered in the organic layer of the THP-1 cell extraction process under negative ion detection mode. Control: untreated cells, PI: cells treated with PI

Neg_Org

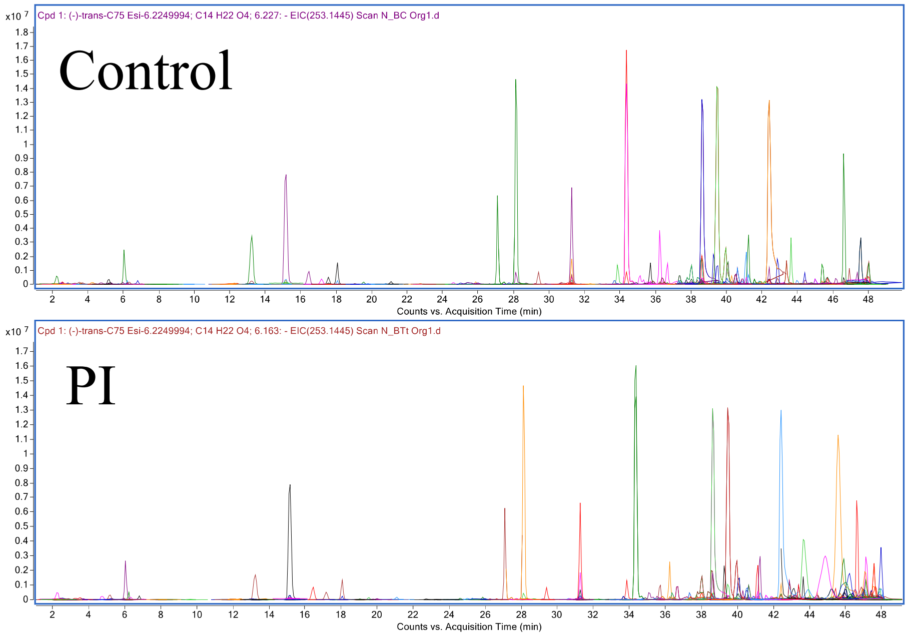

Metabolites recovered in the organic layer of the THP-1 cell extraction process under negative ion detection mode. Butanol: cells treated with n-butanol, the vehicle; PI: cells treated with PI

Neg_Org

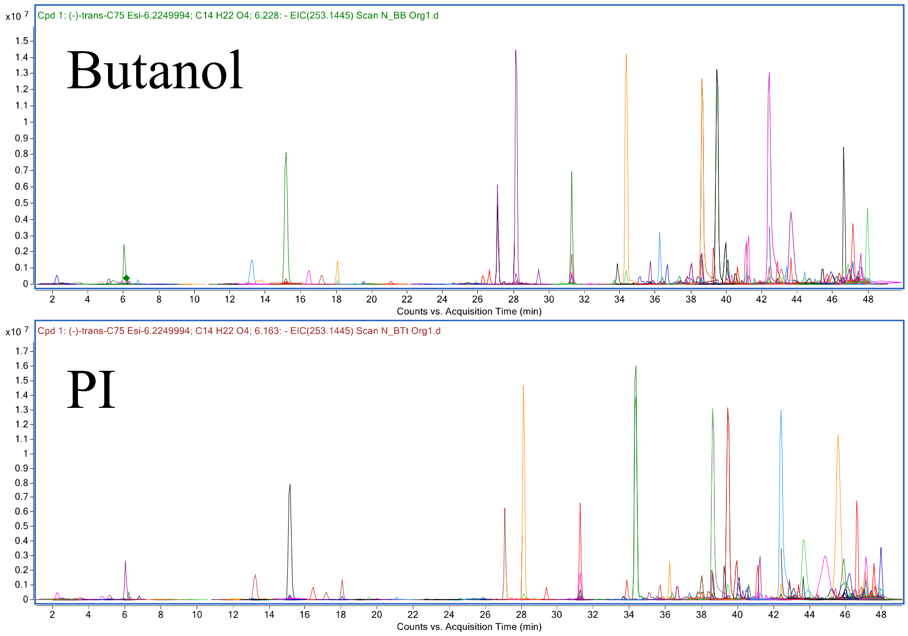

Metabolites recovered in the aqueous layer of the THP-1 cell extraction process under positive ion detection mode. Control: untreated cell; PI: cells treated with PI

Pos_Aq

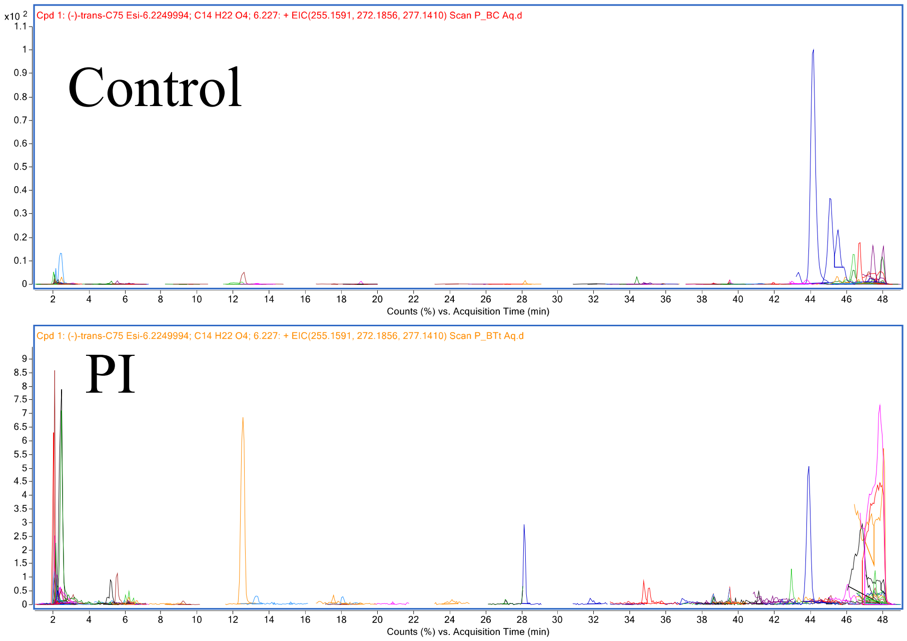

Metabolites recovered in the aqueous layer of the THP-1 cell extraction process under positive ion detection mode. Butanol: Cells treated with n-butanol, the vehicle; PI: cells treated with PI

Pos_Aq

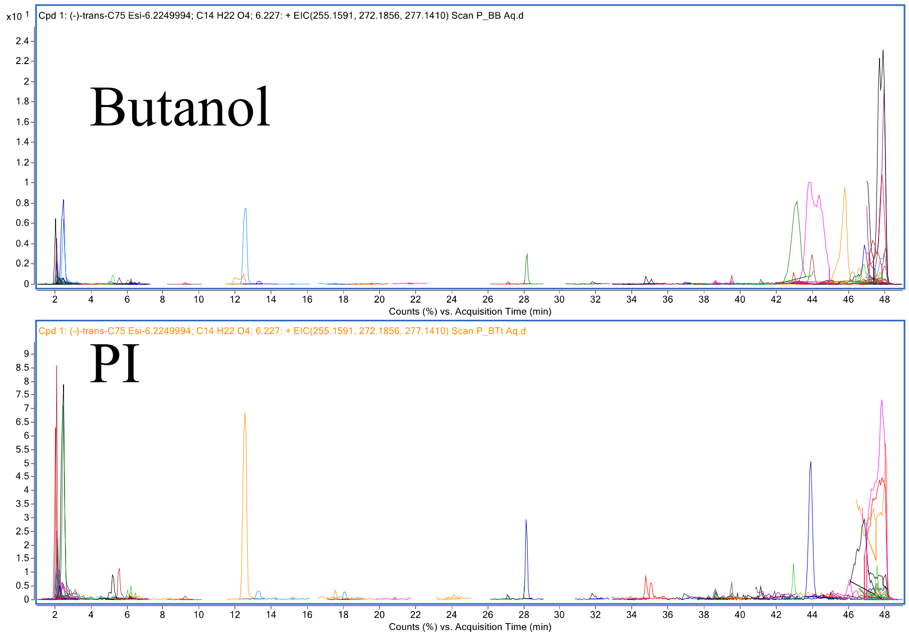

Metabolites recovered in the organic layer of the THP-1 cell extraction process under positive ion detection mode. Control: untreated cell; PI: cells treated with PI

Pos_Org

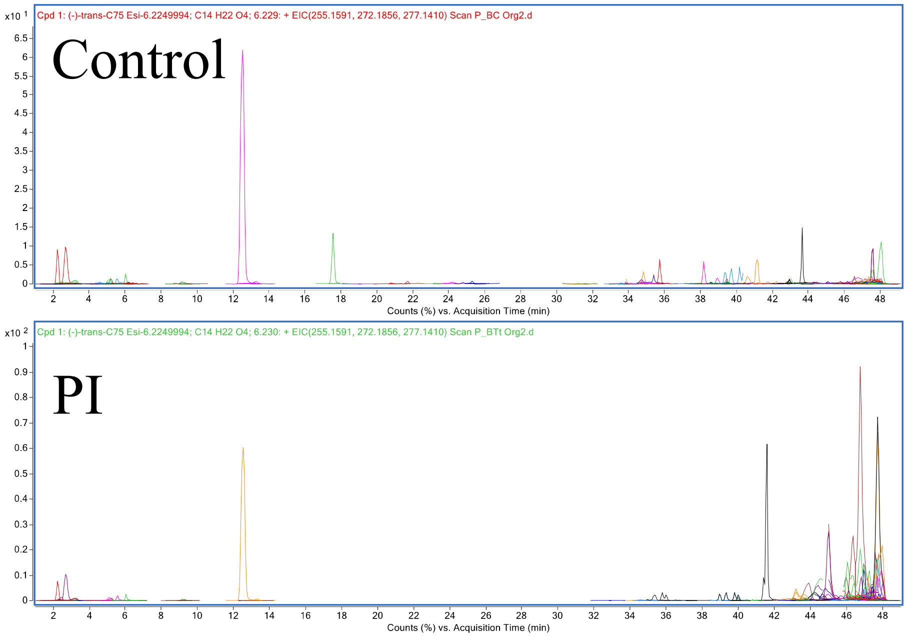

Metabolites recovered in the organic layer of the THP-1 cell extraction process under positive ion detection mode. Butanol: Cells treated with n-butanol, the vehicle; PI: cells treated with PI

Pos_Org

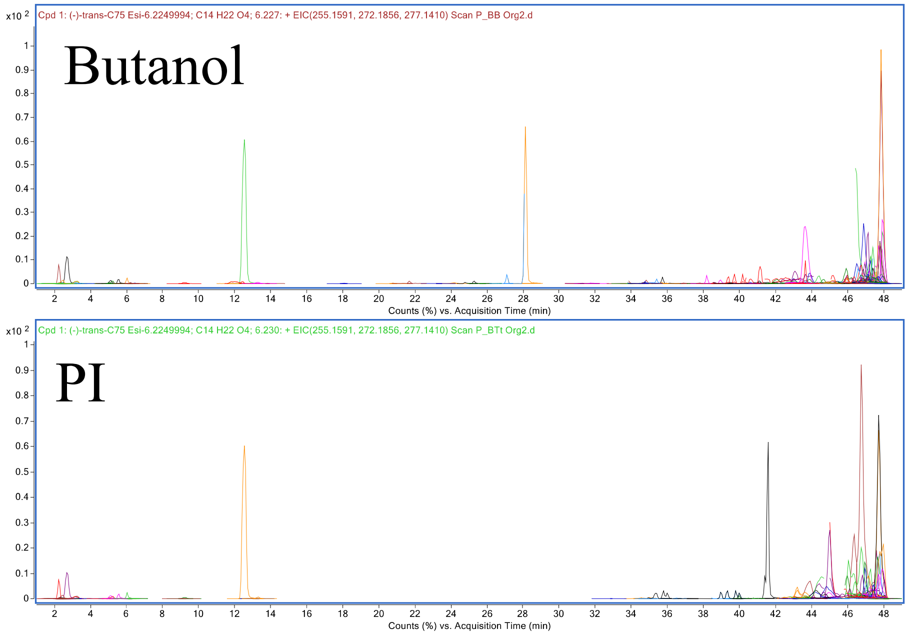

Figure S2. Flow Cytometry data of immunophenotyping **whole blood, bone marrow and splenic immune cells.**

A. Flow cytometry data used to generate Figure 6B.

Polyprenyl immunostimulant (PI) does not significantly impact bone marrow HSPC frequencies.

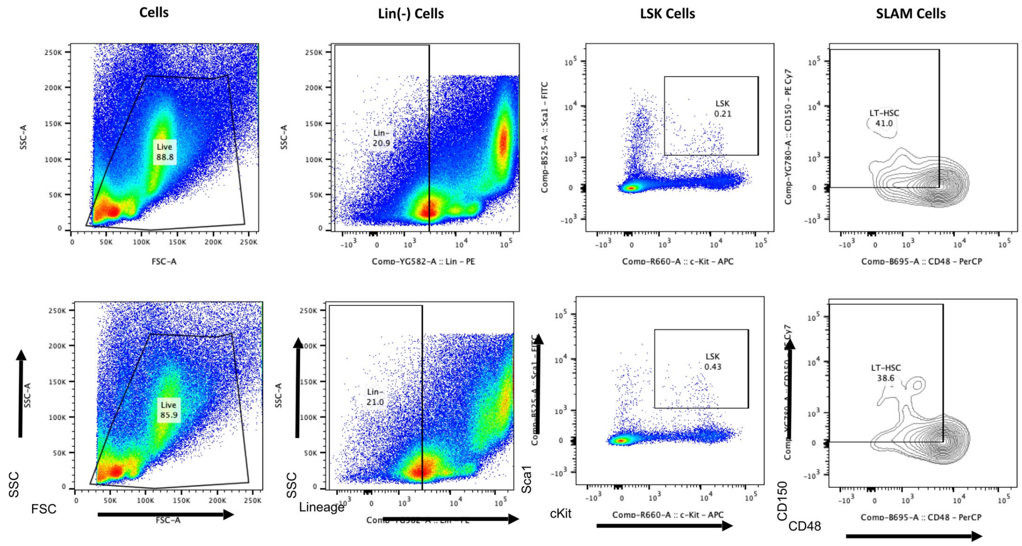

Polyprenyl immunostimulant (PI) does not significantly impact splenic HSPC frequencies.

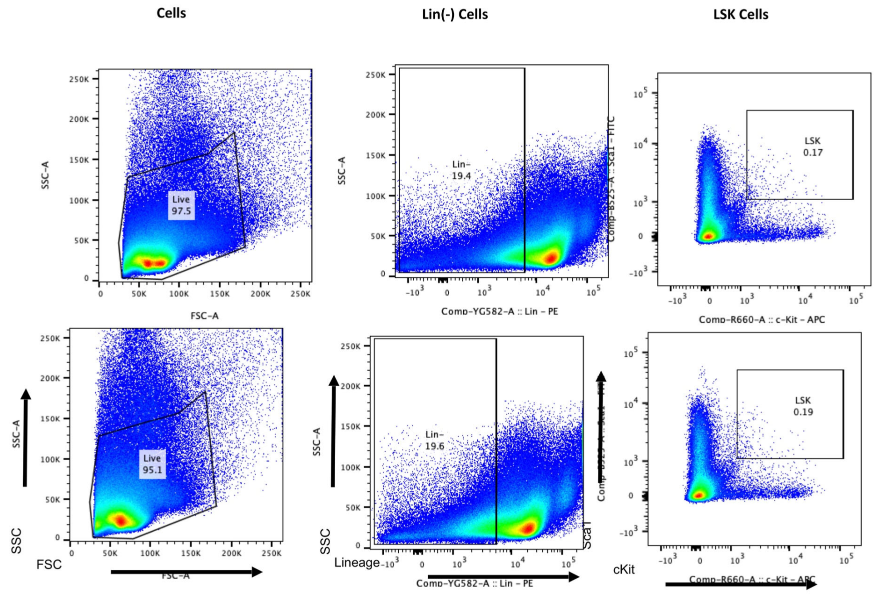

B. Flow cytometry data used to generate Figure 6C.

Polyprenyl immunostimulant (PI) reduces bone marrow B-cells and alters bone marrow T-cell frequencies

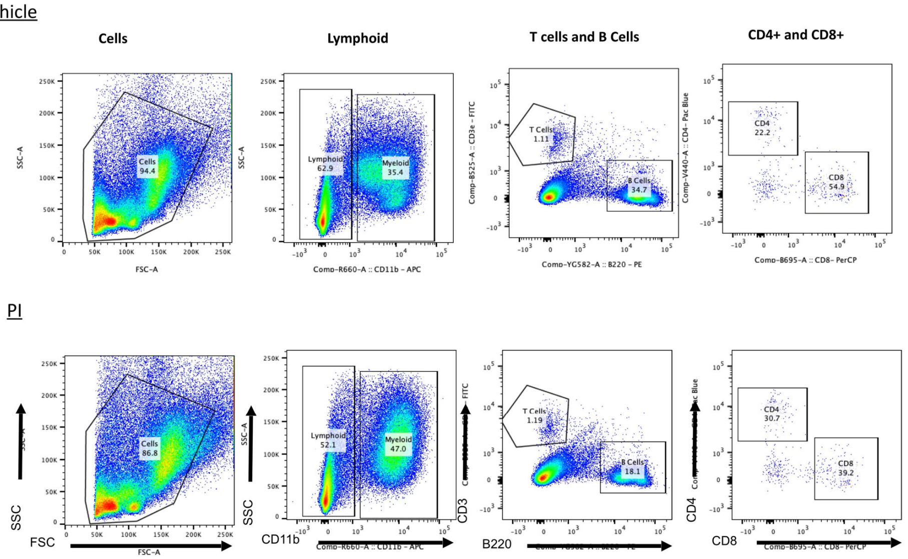

C. Cytometry data used to generate Figure 6D.

Polyprenyl immunostimulant (PI) alters splenic T-cell but not B-cell frequencies

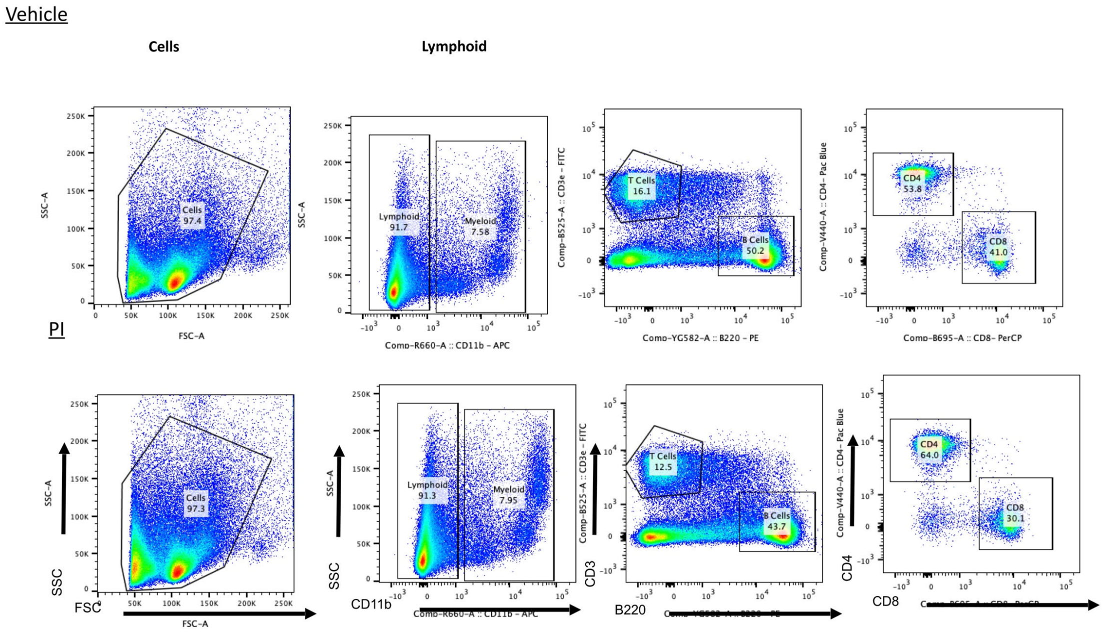

Supplementary Data

Common metabolites that differ by 2-fold, found in PI treated THP-1 and mice blood cells are supplied in the excel file.

Metabolites involved in the sphingolipid metabolism are highlighted in yellow.
